# Procedure rates performed by emergency medicine residents: a retrospective review

**DOI:** 10.1186/s12245-018-0167-x

**Published:** 2018-02-14

**Authors:** Joshua T. Bucher, Christopher Bryczkowski, Grant Wei, Renee L. Riggs, Anoop Kotwal, Brian Sumner, Jonathan V. McCoy

**Affiliations:** 1 Department of Emergency Medicine, Rutgers Robert Wood Johnson Medical School, 1 Robert Wood Johnson Pl. MEB Rm. 104, New Brunswick, NJ 08903 USA; 2 George Washington University Medical School, Washington D.C, USA; 3 Jersey Shore University Medical Center, Neptune City, NJ USA

**Keywords:** Procedure rates, Residency education, Procedural competency, ACGME, Education, Resident education, Procedures

## Abstract

**Background:**

The purpose of our study is to investigate rates of individual procedures performed by residents in our emergency medicine (EM) residency program. Different programs expose residents to different training environments. Our hypothesis is that ultrasound examinations are the most commonly performed procedure in our residency.

**Methods:**

The study took place in an academic level I trauma center with multiple residency and fellowship programs including surgery, surgical critical care, trauma, medicine, pulmonary/critical care, anesthesiology and others. Also, the hospital provides a large emergency medical services program providing basic and advanced life support and critical care transport, which is capable of performing rapid sequence intubation. Each EM residency class, except for the first 2 months of the inaugural class, used New Innovations to log procedures. New Innovations is an online database for tracking residency requirements, such as procedures and hours. For the first 3 months, procedures were logged by hand on a log sheet. In addition, our department has a wireless electronic system (Qpath) for recording and logging ultrasound images. These logs were reviewed retrospectively without any patient identifiers.

Actual procedures and simulation procedures were combined for analysis as they were only logged separately halfway through the study period. Procedures were summed and the average procedure rate per resident per year was calculated.

**Results:**

In total, 66 full resident years were analyzed. Overall, ultrasound was the most commonly performed procedure, with each resident performing 125 ultrasounds per year. Removing “resuscitations,” the second most common was endotracheal intubation, performed 28.91 times per year, and third most was laceration repair, which was performed 17.39 times per year.

Our lowest performed procedure was thoracentesis, which was performed on average 0.11 times per resident per year.

**Conclusions:**

Residents performed a variety of procedures each year. Ultrasound examinations were the most frequent procedure performed. The number of ultrasound procedures performed may reflect the changing training landscape and influence future Accreditation Council of Graduate Medical Education requirements.

## Background

Emergency medicine (EM) residents are expected to perform certain numbers of procedures in order to obtain procedural competence as directed by the Accreditation Council for Graduate Medical Education (ACGME) and the emergency medicine Residency Review Committee (RRC). While programs routinely report this data to the RRC, to our knowledge, there are no recently published studies in the literature that have looked at overall emergency medicine resident procedure rates, with the two identified studies published in 1998 and 1999 [1, 2]. Our goal is to publish data for other residency programs to use as a benchmark.

Our study is designed to compare rates of various procedures that were entered into our emergency medicine resident’s procedures logs. Specifically, our single center information will help plan future education focus and identify opportunities for improvement as residency training has changed in the almost two decades that have lapsed since those two studies were published [1, 2]. More generally, as other institutions also add to the literature, information on procedure rates can influence the developmental milestones that are currently being used to track resident educational progress but are in flux due to lack of adequate transparent data [3].

While procedural competency is a required part of residency education, there is little published data on the number of required procedures in order to obtain “competency.” For example, the RRC lists 35 endotracheal intubations are required to obtain adequate “competency” on their evaluation. However, there is data from the anesthesia literature that suggests this number is closer to 200 intubations before true procedural competency is achieved [4]. Our data will contribute our numbers to the literature for comparison.

## Methods

We performed a retrospective review of all resident procedure logs of the emergency medicine resident physicians at our institution. The emergency medicine residency program at our institution is an ACGME-accredited, 3-year program that consists of six residents per year and enrolled its first class in 2010. All resident classes were included prior to the beginning of the class of 2018 which started in July 2015. Two residents of the first 18 did not complete a full year, and their data was excluded from analysis. No other data was excluded. Three classes of residents graduated from the program since inception and were included in their entirety. One class had 2 years of data and one class had only 1 year of data; both classes were included.

The study was performed at an academic, urban, tertiary care center; comprehensive stroke center; level one trauma center; and cardiac catheterization center located in the Northeast. Our emergency department has a volume of approximately 70,000 adult visits per year and 20,000 pediatric visits in the dedicated pediatric emergency department.

Three different systems were used to collect data. In the first few months of the program, procedure logs were recorded by hand by the residents. Since then, a commercial, web-based, HIPPA compliant program, New-Innovations.com, has been utilized. It tracks patient encounters and procedures logged by the resident physician and verified by the attending who supervised the procedure. Lastly, QPath, a commercial, web-based bedside ultrasound program, was used to record ultrasound examinations performed by residents.

After all patient identification was removed from the records, we received a list of total number of procedures per resident, per year. The data was combined and recorded into an Excel spreadsheet, and basic statistics were used to describe the numbers of procedures.

Our research protocol was approved by our medical school Institutional Review Board.

## Results

Our results demonstrate that overall, the most common emergency procedure performed by residents was bedside ultrasonography. On average, each resident performed 125 ultrasound examinations per year of residency. Likewise, it was found to be the largest performed procedure in terms of overall metrics, with one resident having logged 684 ultrasounds upon graduation.

Conversely, thoracentesis was the least likely performed procedure overall. On average, residents performed 0.11 thoracentesis yearly and only one resident had logged more than one in their logs. Furthermore, intraosseous access was the second least performed procedure at only 0.2 per resident/year and a maximum of two were logged for any one resident. A complete summary of total numbers of each procedure including the average per resident year and the maximum performed by any single resident can be found in Tables 1 and 2.

**Table 1:** Total and average number of procedures (including simulation) per resident per year (i.e., the number of procedures one resident will perform each year of residency)

Procedure	Total	Average
Arterial line	317	4.8
Arthrocentesis	71	1.08
Cardiac pacing	142	2.15
Cardioversion	281	4.26
Central venous access	1102	16.7
Chest tube	317	4.8
Cricothyrotomy	68	1.03
Dislocation reduction	399	6.05
I&D	235	3.56
Intraosseous access	13	0.2
Intubation	1908	28.91
Laceration repair	1147	17.39
Lateral canthotomy	47	0.71
Lumbar puncture	393	5.95
Paracentesis	26	0.39
Pericardiocentesis	49	0.73
Procedural sedation	379	5.74
Regional anesthesia	153	2.32
Adult medical resuscitation	3886	58.88
Adult trauma resuscitation	1217	18.44
Pediatric medical resuscitation	596	9.03
Pediatric trauma resuscitation	305	4.62
Splinting	468	7.09
Thoracentesis	7	0.11
Thoracotomy	51	2.55
Vaginal delivery	180	5.35
Ultrasound	8252	305.85

**Table 2:** The maximum number of procedures performed by one individual resident over their residency

Procedure	Maximum
Arterial line	34
Arthrocentesis	7
Cardiac pacing	16
Cardioversion	28
Central venous access	76
Chest tube	27
Cricothyrotomy	5
Dislocation reduction	33
I&D	25
Intraosseous access	2
Intubation	157
Laceration repair	105
Lateral canthotomy	4
Lumbar puncture	25
Paracentesis	5
Pericardiocentesis	5
Procedural sedation	32
Regional anesthesia	21
Adult medical resuscitation	406
Adult trauma resuscitation	80
Pediatric medical resuscitation	59
Pediatric trauma resuscitation	23
Splinting	73
Thoracentesis	2
Thoracotomy	5
Vaginal delivery	21
Ultrasound	684

## Discussion

There is limited data on the optimum number of procedures emergency medicine residents need to perform to develop competency. The ACGME has set requirements for emergency medicine residents to perform specific procedures [3]. However, these numbers appear to be based on the expert opinion of the Residency Review Committee rather than literature derived.

On average, each resident performed 29 (range 9–52) intubations per year. Thus, our residents quickly met the 35 endotracheal intubation target requirement set by the ACGME. Although the purpose of this cross-sectional study was to provide a snapshot in time of procedure numbers, this result in particular begets the question how many procedures beyond the requirement lead to proficiency? Is 35 intubations per resident an adequate number? Does it mean adequate actual intubation skill or the recognition of difficult airways where advanced procedures are necessary or both? Is there clear learning benefit by more than doubling the requirement as in our program? Sagarin et al. found that as resident physicians increased in year of residency training, their success at intubation attempts improved [5].

Furthermore, video laryngoscopy has become a frequently available tool to complete the procedure. Nevertheless, it is important that residents obtain proficiency in video laryngoscope as well as direct laryngoscopy. We do not track video and direct laryngoscopy separately. How many intubations with and without video laryngoscopy assistance is necessary for proficiency also remains unclear. Sylvia et al. found that emergency medicine residents and pediatric residents had no difference in intubation success using video laryngoscopy in a simulated pediatric airway model, and overall, success was poor [6]. Perhaps a separate skill, video laryngoscopy, needs to be considered as an additional procedural competency by the ACGME for emergency medicine residents in addition to the minimum number of traditional direct laryngoscopy intubations [7].

Bernhard et al. studied intubation success in a prospective observation trial of German anesthesia residents. They compared intubation success and complications for every 25 intubations until each trainee had achieved 200 intubations and found that first past success increased (67 vs 83%, *p* = 0.0001) and total success and number of attempts required (1.6 vs 1.3, *p* = 0.0001) significantly improved by the time the trainee had amassed 200 intubations [4]. They concluded that their results should potentially influence the requirements for anesthesia and emergency medicine requirements. While this study was not performed on emergency medicine residents, nor was it performed in the USA, endotracheal intubation is a critical procedure that requires the best probability of first pass success, and our training requirements should reflect that. Moreover, just as importantly, some may question how many intubations are required to recognize procedural competency. This may have even more significance for EM residents and physicians.

On average, each resident performed six lumbar punctures a year. This just exceeds the minimum requirement of 15 procedures. Lammers et al. studied new postgraduate year (PGY) 1 resident proficiency in performing a lumbar puncture on a standard model by judging specific steps that needed to be performed [8]. Although 83% of PGY 1 EM residents reported having had performed the procedure on a patient during medical school, they found that PGY 1 residents had not attained competence. During the study, 69% of the attempts resulted in a failure to obtain cerebrospinal fluid. These findings suggest that more training in lumbar puncture may be beneficial.

Ultrasound numbers also proved interesting, with the average resident in our program performing and documenting over 370 scans in the various modalities, averaging 125 per year. This far exceeds the milestone 4 threshold of 150 ultrasounds, though meeting that number as a sole marker of competence has already been questioned, and a more nuanced pathway towards competence has been published [9]. There have been various studies published advocating or proposing competence with different numbers of studies for different ultrasound studies, varying from as little as 10 ultrasounds for FAST [10, 11] to 25–30 for gallbladder studies [12].

Each resident performed many adult medical and adult trauma resuscitations, averaging 59 and 18 a year respectively. The milestones delve deeper into resident competence, but the raw number of resuscitations does not measure team participation versus team leadership and developing a command presence, which are emphasized in the competency milestone.

There were many procedures that were performed infrequently, including arthrocentesis, pacing, lateral canthotomy, thoracotomy, pericardiocentesis, cricothyrotomy and others.

Thoracentesis is a relatively uncommon procedure performed in an emergency setting, and this, as well as our institutional tendency to defer this procedure to interventional radiology, likely resulted in its finish as the least commonly performed logged procedure. Educational efforts, such as simulation, cadaver lab, and models for these procedures may provide educational benefit.

The high utilization of bedside ultrasound at our program, including its use for peripheral venous access, could have impacted the use of other vascular access adjuncts including intraosseous access, one of the lowest performed procedures in our cohort.

Furthermore, the use of simulation can help to provide experiences to residents in high-risk low-frequency events, such as cardiac arrest or specific cardiovascular emergencies. Okuda reviewed the literature in relationship to resident training and cardiac arrest with the use of simulation and found there is a large variety of options to provide this training to the residents [7]. This type of experience can compliment resident training and can be targeted to lower frequency procedures as identified in our study.

### Limitations

There are several limitations to our study. Our method of recording procedures switched after the first few months of the residency program’s start. Initial handwritten records were available and used for data collection but were less reliable compared to later computerized records due to handwriting and quality of the paper after years of storage.

Our results also include both actual and simulated procedures combined. For the first half of the residency program, simulated procedures were not recorded separately from actual procedures performed on patients. After the first 2.5 years, we began recording simulated procedures separately. Therefore, we kept these data combined for consistency as we had no reliable, efficient method to separate them. This can influence our results, especially for rarely performed procedures such as cricothyrotomy, lateral canthotomy, pericardiocentesis, and thoracotomy.

Furthermore, the study relies on individual residents self-reporting procedures completed. After the resident meets the required number of procedures for graduation, the incentive to continue recording every procedure performed decreases, and there may be a large number of procedures performed later in training that were not recorded. This may underestimate the actual number of procedures performed per resident. For example, the average number of intubations is 28.9 per resident per year (86.7 over 3 years), but the highest number of intubations performed is 157 by a single resident over 3 years, almost twice the average.

Perhaps most significantly, this was a single site study—there is likely significant variation in the types of patients, amount of penetrating vs. blunt trauma, deferral of procedures to other services—depending on the site/institution of the residency program.

## Conclusions

Emergency medicine residents perform many procedures; bedside ultrasound is by far the most frequent. Further investigation into procedural competency and resident self-recognition of limitations is needed.
